# Supplementary material for: Fast and Deep Diagnosis Using Blood-Based ATR-FTIR Spectroscopy for Digestive Tract Cancers
Source: Biomolecules. 2022 Dec 5;12(12):1815. doi: 10.3390/biom12121815 (PMC9775374; doi:10.3390/biom12121815)
Supplement: Supplementary file 1 [file biomolecules-12-01815-s001.zip › biomolecules-1911337-supplementary.pdf]

Supplementary Tables

Supplementary Table S1: Retrospective patient subdivision of cohort with information on DTC classification.

Tumors(n)	Stages	Participants	Specimens	Sex (M/F)	Age range	Average age	Class of ML
LC (25)	Cirrhosis	2	4	2/2	46-50	48	1
	LC stage	10	12	10/2	52-73	63	2
	Postoperative	13	13	8/5	46-81	64	3
GC (68)	Gastritis	4	5	4/1	47-59	53	1
	GC stage	30	34	26/8	47-83	65	2
	Postoperative	34	37	30/7	52-84	68	3
CC (73)	Obstruction	4	7	4/3	45-61	53	1
	CC stage	25	30	16/14	51-78	65	2
	Postoperative	44	66	34/32	51-81	66	3
Control		44	44	22/20	48-83	52	4

Tumors: Liver cancer (LC), gastric cancer (GC) and colorectal cancer (CC); n: total; Class: Grouping of ML based on blood for DTCs.

Supplementary Table S2: SD-IR spectra: Distribution and statistical comparison of DTC and control spectra in terms of band position and relative absorbance.

Centered bands	Band locations/(cm^{-1})			Absorbance (a.u.)		
	LC (*p*)	GC (*p*)	CC (*p*)	LC (%)	GC (%)	CC (%)
3195	3195.8	3196.8(**)	3195.1(*)	0.53(0)	0.56(+5.6)	0.55(+3.7)
3089	3089.3(*)	3089.1(*)	3088.8	0.66(0)	0.66(0)	0.67(+1.5)
2965	2967.9(***)	2965.5(*)	2966.7(**)	0.68(+4.6)	0.65(0)	0.68(+4.6)
2925	2925.9	2924.7(**)	2925.9	0.64(+8.5)	0.62(+5.0)	0.66(+11.9)
2898	2898.4(*)	2899.0	2897.7(**)	0.72(+1.4)	0.73(+2.8)	0.71(0)
2857	2855.7(**)	2856.2(*)	2859.2(***)	0.66(+3.1)	0.63(-1.6)	0.67(+4.7)
1741	1742.6(**)	1740.6	1741.6(**)	0.69(+3.0)	0.68(+1.5)	0.71(+6.0)
1695	1693.4(**)	1698.9(***)	1695.3(***)	0.84(-2.3)	0.84(-2.3)	0.84(-2.3)
1639	1638.2(**)	1639.7	1638.6(**)	0.02(+100.0)	0.07(+600.0)	0.02(+100.0)
1574	1574.9(**)	1574.1(**)	1575.6(*)	0.85(-5.6)	0.88(-2.2)	0.88(-2.2)
1544	1545.9(*)	1544.1(*)	1544.9(*)	0.53(+26.2)	0.41(-2.4)	0.53(+26.2)
1508	1508.1(**)	1508(**)	1508.1(**)	0.74(+4.2)	0.72(+1.4)	0.73(+2.8)
1455	1456.0(*)	1456.3(*)	1455.3	0.63(+3.3)	0.60(-1.6)	0.63(+3.3)
1427	1428.2(*)	1426.5(*)	1427.3	0.72(-2.7)	0.75(+1.3)	0.73(-1.3)
1398	1399.6(*)	1398.6	1399.6(*)	0.64(+6.7)	0.57(-5.0)	0.64(+6.7)
1354	1354.2	1354.1	1354.3	0.69(+1.5)	0.68(0)	0.69(+1.5)
1309	1310.7(**)	1309.7(*)	1310.7	0.68(+6.3)	0.65(+1.6)	0.67(+4.7)
1240	1242.1(***)	1239.4	1241.1(**)	0.67(+4.7)	0.66(+3.1)	0.67(+4.7)
1166	1165.2(*)	1165.2(*)	1166.2	0.67(+1.5)	0.66(0)	0.67(1.5)
1117	1119.4(***)	1119.4(***)	1118.2(***)	0.67(0)	0.67(0)	0.67(0)
1082	1082.6(***)	1080.8(**)	1082.7(***)	0.69(+3.0)	0.65(-3.0)	0.68(+1.5)
1040	1041.9(***)	1039.7	1041.2(**)	0.68(+3.0)	0.69(+4.5)	0.68(+3.0)
986	984.8	986.1(**)	986.1(**)	0.70(+2.9)	0.69(+1.5)	0.70(+2.9)

Tumors: Liver cancer (LC), gastric cancer (GC) and colorectal cancer (CC); Changes (%) are compared to controls in average band absorbance; *p<0.05, **p<0.01, ***p<0.001.

Supplementary Table S3: Accuracy of results obtained using multiple multivariate methods.

Cancers	Methods	Accuracies of classification			
		IR Absorbance(%)	IR Absorbance +shift(%)	SD-IR Absorbance(%)	SD-IR Absorbance +shift(%)
LC	BP	70.60 (±0.44)	89.40 (±0.23)	76.90 (±0.23)	95.60 (±0.06)
	KNN	81.75 (±0.33)	89.85 (±0.18)	83.87 (±0.12)	93.00 (±0.09)
	RF	75.00 (±0.41)	83.75 (±0.22)	80.65 (±0.17)	86.25 (±0.13)
	DT	83.75 (±0.25)	89.87 (±0.14)	88.75 (±0.12)	92.50 (±0.07)
	Logistic	65.00 (±0.49)	75.00 (±0.22)	70.00 (±0.21)	83.25 (±0.12)
	SVM	68.75 (±0.36)	86.25 (±0.21)	86.87 (±0.35)	94.75 (±0.02)
	MVLR	78.86 (±0.24)	82.43 (±0.19)	87.40 (±0.23)	89.20 (±0.13)
	PLS-DA	82.30 (±0.16)	87.20 (±0.12)	88.25 (±0.21)	91.47 (±0.05)
GC	BP	75.00 (±0.22)	85.20 (±0.15)	77.50 (±0.33)	98.70 (±0.06)
	KNN	78.75 (±0.23)	79.00 (±0.28)	82.50 (±0.25)	96.87 (±0.02)
	RF	72.25 (±0.31)	78.75 (±0.31)	75.63 (±0.21)	88.13 (±0.12)
	DT	74.30 (±0.26)	84.13 (±0.24)	83.00 (±0.15)	92.50 (±0.09)
	Logistic	58.75 (±0.45)	85.00 (±0.12)	80.62 (±0.14)	89.25 (±0.11)
	SVM	46.88 (±0.49)	76.25 (±0.29)	84.50 (±0.12)	93.12 (±0.07)
	MVLR	77.20 (±0.11)	84.25 (±0.17)	86.45 (±0.09)	94.75 (±0.02)
	PLS-DA	79.50 (±0.13)	85.20 (±0.15)	92.20 (±0.01)	98.42 (±0.01)
CC	BP	65.60 (±0.33)	70.60 (±0.24)	69.40 (±0.24)	95.00 (±0.12)
	KNN	69.37 (±0.29)	72.50 (±0.27)	74.87 (±0.28)	90.25 (±0.02)
	RF	61.80 (±0.38)	69.87 (±0.29)	81.25 (±0.25)	91.00 (±0.12)
	DT	65.00 (±0.33)	73.12 (±0.26)	78.87 (±0.27)	92.50 (±0.10)
	Logistic	56.87 (±0.41)	68.12 (±0.31)	68.13 (±0.32)	78.75 (±0.21)
	SVM	55.00 (±0.43)	56.25 (±0.34)	79.65 (±0.23)	96.85 (±0.05)
	MVLR	69.50 (±0.28)	74.50 (±0.20)	80.74 (±0.19)	91.13 (±0.09)
	PLS-DA	70.50 (±0.14)	76.30 (±0.17)	82.20 (±0.18)	92.75 (±0.04)

Supplementary Figures

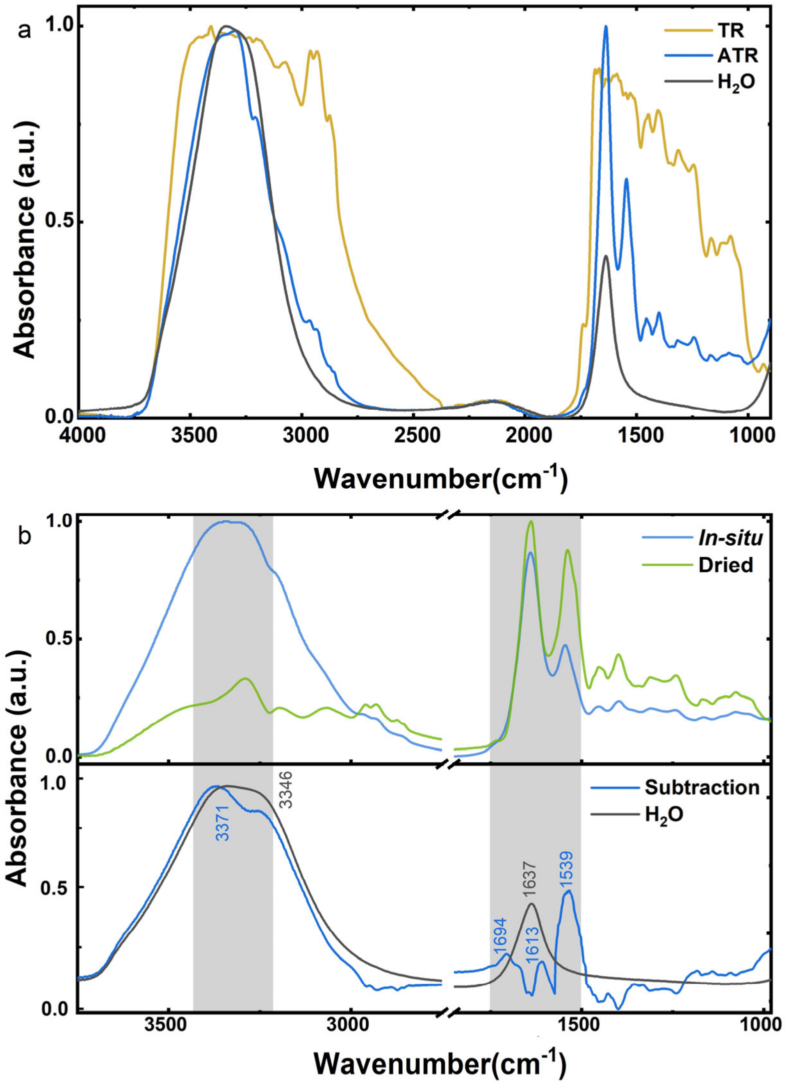

Supplementary Figure S1*:* Optimization of FTIR experimental technical route. (a) Selection of the suitable ATR-FTIR mode by comparing the results of transmission (TR) and attenuated total reflection (ATR) measurement modes. (b) Spectral comparison of the *in-situ* and dried serum samples and the difference between the *in-situ* and dried serum sample spectra (subtraction) was compared with pure water samples. Our testing results demonstrated that ATR-FTIR was more suitable for liquid biofluids than TR-FTIR with heavy flat and noisy peaks, and dehydration of sample caused the lack of *in-situ* information, not only H_2O.

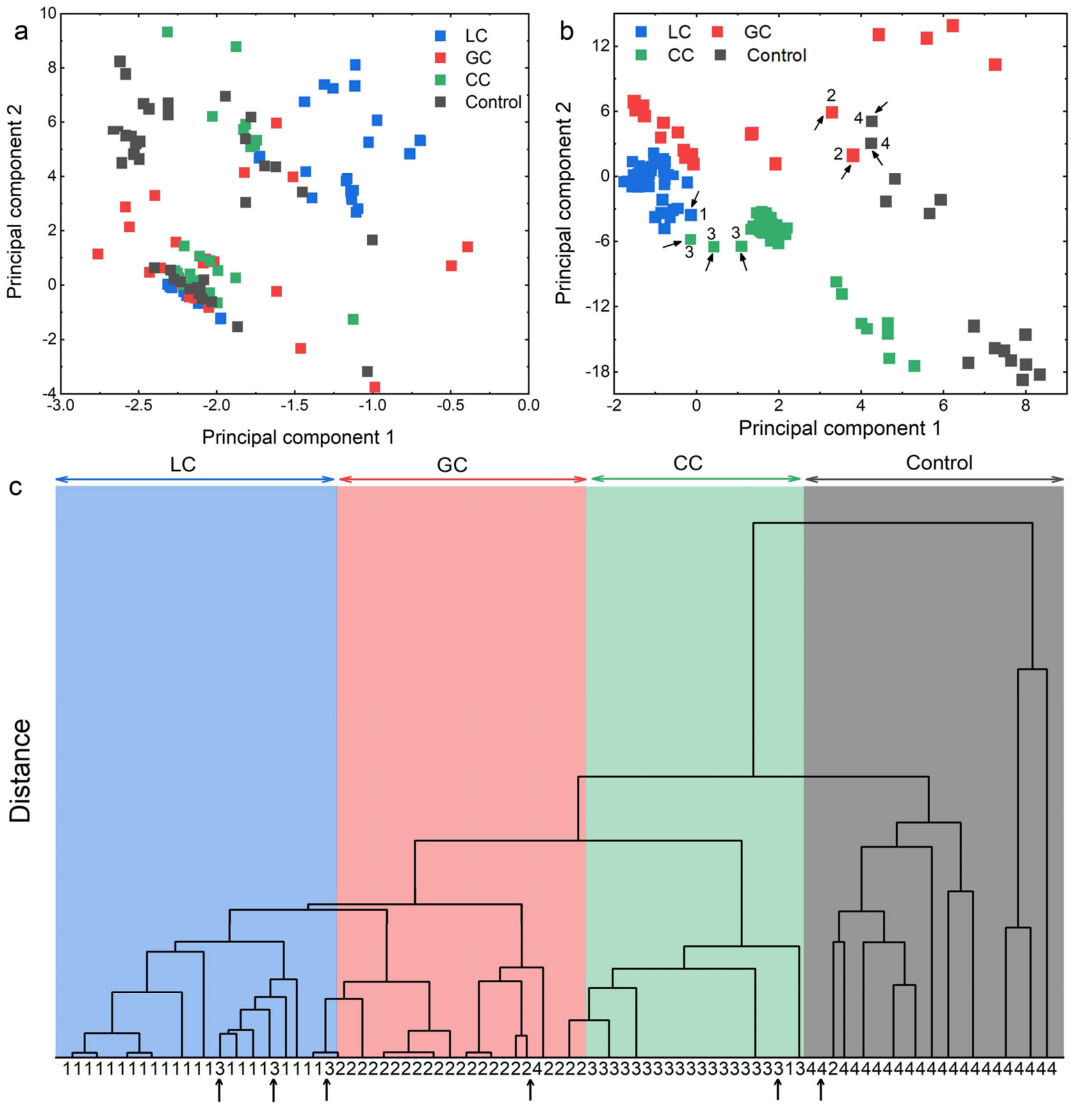

Supplementary Figure S2: Due to the similarity of the normalized serum-based attenuated total reflection mode of FTIR (ATR-FTIR) spectra in the range of 900-3500 cm^{-1}, principal component analysis (PCA) downscaling was performed to identify serum samples of DTCs. In terms of PCA classification, the first two PCAs were used for multivariate data sets, and their fractional plots showed different classification results, as shown in (a, b). It could be seen that the infrared spectra (SD-IR) based PCA classification was outperformed the infrared spectroscopy (IR)-based classification, which was almost unidentifiable in (a). The DTC serum samples were virtually discerned with the exception of a very limited amount of data points shown by arrows in (b). Hierarchical cluster analysis (HCA) classification of DTC motifs was carried out using the minimum distance method and the Euclidean distance based on second derivative SD-IR. Most of the DTC samples were accurately clustered, except for only six misclassifications, shown in (c). This indicated that PCA and HCA had the ability of both unsupervised methods to classify DTCs before blood-based tests, and that HCA performs slightly better than PCA. The results of the two unsupervised methods showed the potential feasibility of ATR-FTIR to identify patients with different DTCs, while the supervised learning method with labeled datasets may provide more profound information. Corresponding to these three groups filled with different colors. 1 = LC; 2 = GC; 3 = CC; 4 = controls.

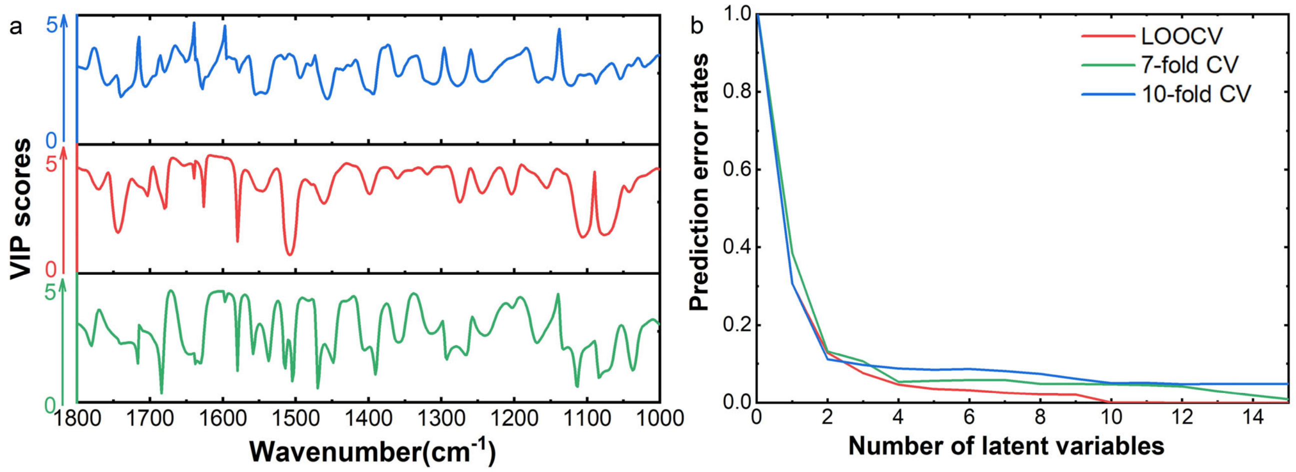

Supplementary Figure S3: (a) Variable importance in projection (VIP) score plot. (b) Error bars were made for the prediction error rate as a function of the number of latent variables. Three most frequent cross-validation (CV) methods: leave-one-out (LOO), 7-fold and 10-fold were used to determine the optimum number of latent variables by means of the prediction error rate.

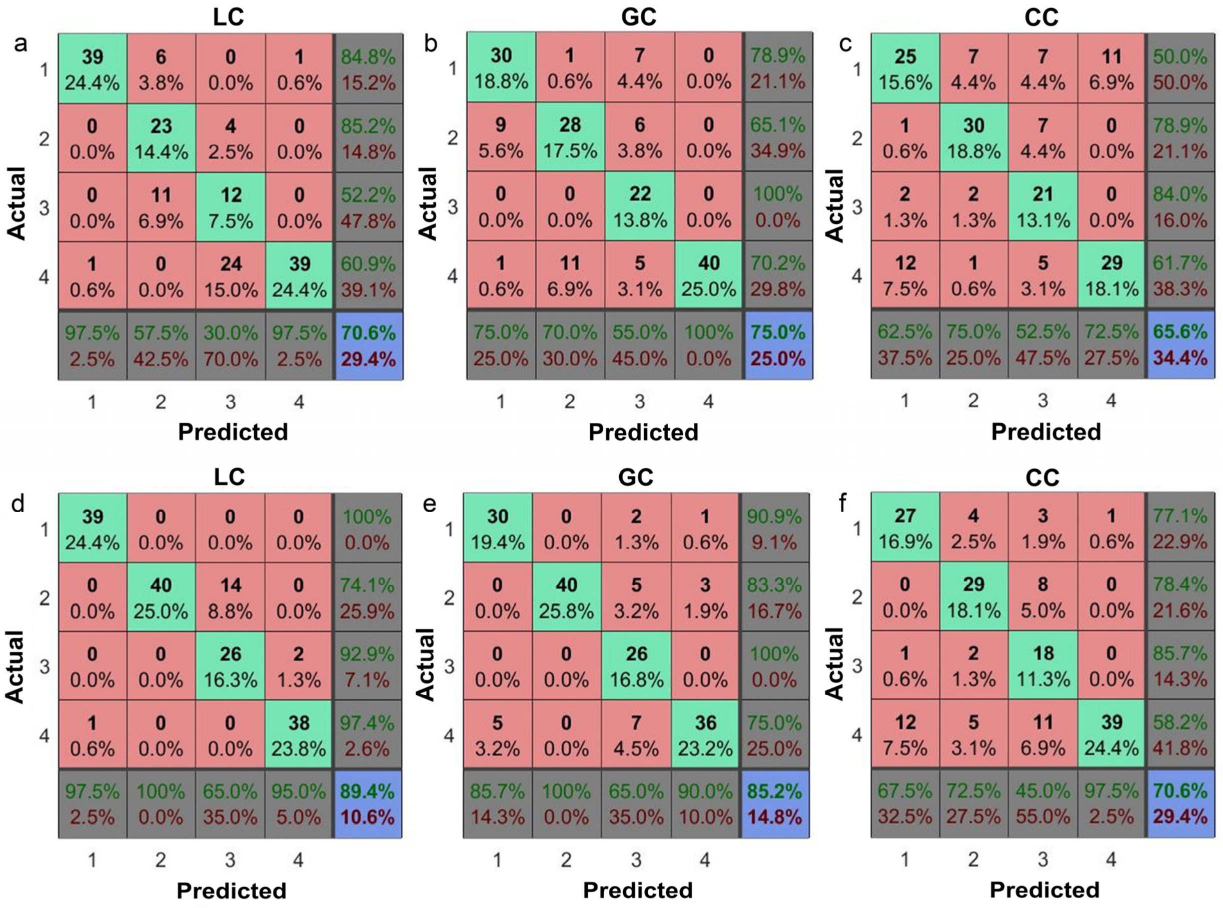

Supplementary Figure S4: Confusion matrix of the prediction accuracy for IR-based blood of DTC stages. (a-c) Prediction reports for the IR absorbance feature dataset from patients with DTCs. (d-f) Prediction results of the optimized IR absorbance + shift feature dataset. Class 1-4: represent different staging of patients related to DTCs detailed seen in Supplementary Table S1, respectively.
